# A Novel off-the-Shelf Trastuzumab-Armed NK Cell Therapy (ACE1702) Using Antibody-Cell-Conjugation Technology

**DOI:** 10.3390/cancers13112724

**Published:** 2021-05-31

**Authors:** Hao-Kang Li, Ching-Wen Hsiao, Sen-Han Yang, Hsiu-Ping Yang, Tai-Sheng Wu, Chia-Yun Lee, Yan-Liang Lin, Janet Pan, Zih-Fei Cheng, Yan-Da Lai, Shih-Chia Hsiao, Sai-Wen Tang

**Affiliations:** Acepodia Biotech Inc., San Mateo, CA 94402, USA; stephanie@acepodiabio.com (C.-W.H.); samshyang@acepodiabio.com (S.-H.Y.); cindy@acepodiabio.com (H.-P.Y.); ericwu@acepodiabio.com (T.-S.W.); sylvia@acepodiabio.com (C.-Y.L.); yanliang@acepodiabio.com (Y.-L.L.); janet@acepodiabio.com (J.P.); zihfei@acepodiabio.com (Z.-F.C.); yanda@acepodiabio.com (Y.-D.L.); sonny@acepodiabio.com (S.-C.H.)

**Keywords:** natural killer cells, oNK, antibody-cell conjugation, ACE1702, cancer cell therapy, HER2, solid tumor

## Abstract

**Simple Summary:**

Chimeric antigen receptor T cell therapy has shown its potency against hematologic malignancies in autologous settings but also limited success against solid tumors with severe adverse events, including fatal cases of cytokine releasing syndrome. The aim of this research is to develop a novel off-the-shelf natural killer cell therapy against HER2-expressing cancers using Antibody-Cell Conjugation (ACC) technology and the endogenous CD16-expressing oNK cell line. ACE1702, trastuzumab-armed oNK cells with γ irradiation and cryopreservation, present superior in vitro and in vivo potency against HER2-expressing cancer cells and shows no tumorigenic potential, indicating the clinical application fighting HER2-expressing solid tumors. These findings suggest that ACC technology can be applied to allogeneic immune cells to provide off-the-shelf therapies for cancer patients.

**Abstract:**

Natural killer (NK) cells harbor efficient cytotoxicity against tumor cells without causing life-threatening cytokine release syndrome (CRS) or graft-versus-host disease (GvHD). When compared to chimeric antigen receptor (CAR) technology, Antibody-Cell Conjugation (ACC) technology has been developed to provide an efficient platform to arm immune cells with cancer-targeting antibodies to recognize and attack cancer cells. Recently, we established an endogenous CD16-expressing oNK cell line (oNK) with a favorable expression pattern of NK activation/inhibitory receptors. In this study, we applied ACC platform to conjugate oNK with trastuzumab and an anti-human epidermal growth factor receptor 2 (HER2) antibody. Trastuzumab-conjugated oNK, ACE-oNK-HER2, executed in vitro and in vivo cytotoxicity against HER2-expressing cancer cells and secretion of IFNγ. The irradiated and cryopreserved ACE-oNK-HER2, designated as ACE1702, retained superior HER2-specific in vitro and in vivo potency with no tumorigenic potential. In conclusion, this study provides the evidence to support the potential clinical application of ACE1702 as a novel off-the-shelf NK cell therapy against HER2-expressing solid tumors.

## 1. Introduction

In recent years, the potential of immune cells for the development of cancer immunotherapy has been realized, including the success of chimeric antigen receptor T cell (CAR-T) therapies, Kymriah and Yescarta, for treatment of acute lymphoblastic leukemia and non-Hodgkin’s lymphoma [1,2]. Although trials of T cells expressing CAR against CD19^+^ lymphoma have demonstrated their efficacy [3,4], T cells expressing CARs against solid tumors have shown limited success [5,6,7]. Accumulating cases of severe adverse events in CAR-T treatment were reported, including fatal cases of cytokine releasing syndrome (CRS) [8,9,10]. Compared to CAR-T, autologous or allogeneic NK cells, including cord blood-derived NK cells and a human NK-92 cell line, possess efficient cytotoxicity against liquid and solid tumors [11,12]. The lack of clonal expansion protects patients from an immune-mediated rejection of allogeneic NK cells. Therefore, severe side effects such as cytokine release syndrome (CRS) or graft-versus-host disease (GvHD) are less likely to occur [11,12,13,14].

Autologous NK cell therapies have not yet shown notable therapeutic effects, mainly due to the difficulty of expanding cells derived from heavily treated patients and the inhibition of self-human lymphocyte antigen (HLA) [15,16]. To overcome the limitation of autologous NK cells, ex vivo expanded allogeneic NK cells provide a sustainable and effective source in clinical applications [15]. In addition to the well-established NK-92 cell line [17,18,19], umbilical cord blood with proper HLA-type selection can be a source of allogeneic NK cells [20,21]. It is also feasible to isolate haploidentical and unrelated donor CD34^+^ progenitor cells or patient HLA-matched NK cells by leukapheresis [22,23]. More recently, controlled differentiation of induced pluripotent stem cells (iPSC) has been shown to provide another source of allogeneic NK cells [24].

We developed the Antibody-Cell Conjugation (ACC) platform to modify cell surfaces with single-strand DNA (ssDNA); the modified cells can be further annealed with the complementary strand-modified molecules [25]. The ACC platform has been applied to link cytokine-induced killer cells (CIKs) with rituximab, while the rituximab-conjugated CIKs have exhibited significant cytotoxicity against CD20-expressing cancer cells [26].

In a recent study, we established a novel endogenous CD16-expressing oNK cell line (oNK) with antibody-dependent cellular cytotoxicity (ADCC), as well as a preferential expression of NK activation receptors and low level of inhibitory receptors [27]. Here, we applied the ACC platform to conjugate trastuzumab on oNK cells and demonstrated its superior in vitro and in vivo potency against HER2-expressing cancer cells. The final product ACE1702, cryopreserved irradiated trastuzumab-conjugated oNK cells, suppresses HER2-expressing cancer cells in vitro and in vivo, and shows no tumorigenicity. This study provides evidence to promote ACE1702 as a potent and safe off-the-shelf cell therapy against HER2-expressing cancers.

## 2. Results

### 2.1. Trastuzumab Conjugation by ACC Technology on oNK Cells

**Figure 1:**
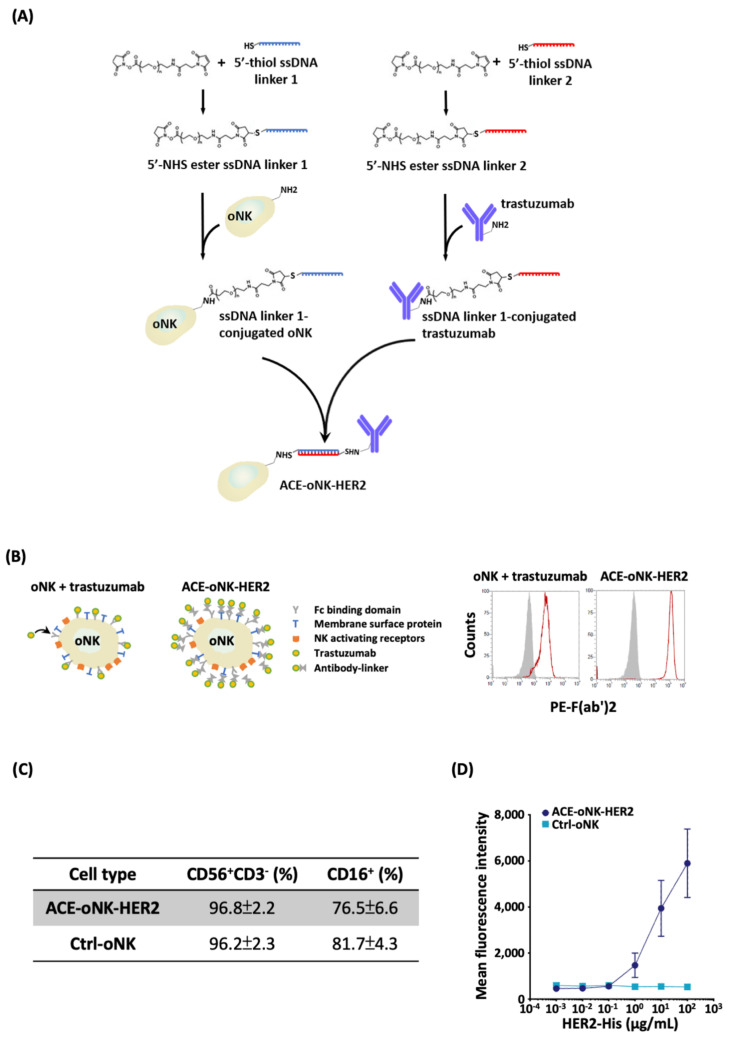
Trastuzumab conjugation confers oNK cells with HER2-specific binding capacity. (**A**) A flow chart of trastuzumab conjugation with oNK cells by ACC technology was illustrated. Preparation of ssDNA linker 1-conjugated oNK and ssDNA linker 2-conjugated trastuzumab was illustrated. Trastuzumab-conjugated oNK cells, ACE-oNK-HER2, were generated by co-incubation of ssDNA linker 1-conjugated oNK and ssDNA linker 2-conjugated trastuzumab. The ssDNA linker 1 and 2 were complementary. (**B**) Antibody loading capacity on the surface of oNK cells by ACC technology or Fc domain binding was illustrated (left panel) and examined using fluorescent dye-conjugated anti-F(ab’)2 antibody by flow cytometry (right panel). Negative staining (gray shaded) and trastuzumab on oNK cells (red line) were shown. (**C**) CD56^+^CD3^-^ and CD16^+^ population as well as (**D**) HER2-His recombinant protein binding capacity were analyzed in both ACE-oNK-HER2 and Ctrl-oNK cells by flow cytometry. The binding capacity was determined by incubating ACE-oNK-HER2 and Ctrl-oNK cells with 10^−3^, 10^−2^, 10^−1^, 10^0^, 10^1^ and 10^2^ μg/mL of human HER2-His recombinant protein. Three independent experiments were performed and the representative results were shown. Each data point was performed in triplicate.

To capacitate oNK cells with HER-targeting specificity, ACC technology was applied to conjugate trastuzumab on oNK cells (ACE-oNK-HER2), which provided >10 fold more antibodies on cell surfaces than those by Fc domain binding (oNK + trastuzumab, Figure 1B). CD56 and CD16 have been shown to characterize oNK [27]. The populations of CD56^+^CD3^-^ and CD16^+^ cells were not influenced by trastuzumab conjugation (Figure 1C). In addition, the expression of NKp30, NKp44, NKG2A and killer-cell immunoglobulin receptors (KIRs) had no significant change after trastuzumab conjugation (Figure S1). To examine the specificity of ACE-oNK-HER2 to HER2 protein, the binding capacity of ACE-oNK-HER2 to HER2-His recombinant protein was analyzed by flow cytometry. As shown in Figure 1D, the HER2 protein bound on the surface of ACE-oNK-HER2 was significantly increased in a dose manner compared to Ctrl-oNK.

### 2.2. Enhanced Cytotoxicity of ACE-oNK-HER2 against HER2^+^ Cancer Cells

**Figure 2:**
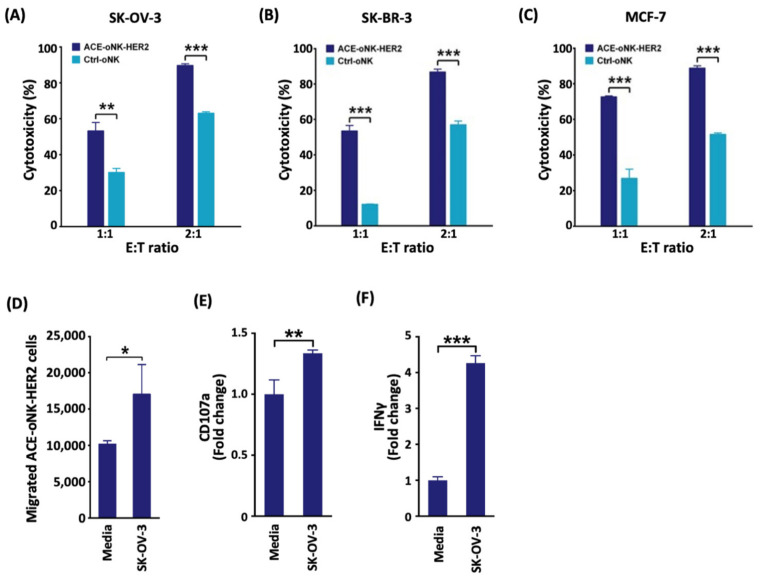
Trastuzumab conjugation confers oNK cells with enhanced cytotoxicity. (**A**–**C**) ACE-oNK-HER2 and Ctrl-oNK were co-incubated with HER2-expressing cancer cells (**A**) SK-OV-3, (**B**) SK-BR-3 and (**C**) MCF-7 at effector to target (E:T) ratio of 1:1 and 2:1 and analyzed by the xCELLigence real-time cell analysis system. (**D**) The migrated ACE-oNK-HER2 in the absence and presence of SK-OV-3 were harvested and stained with anti-CD56 antibody for flow cytometry analysis. (**E**,**F**) ACE-oNK-HER2 was co-incubated with or without SK-OV-3 in the presence of brefeldin A and monensin for 4 h. The cells were harvested, fixed and stained with anti-CD107a or -IFNγ antibody for flow cytometry analysis. Three independent experiments were performed and the representative results were shown. Each data point was performed in triplicate. Statistical analysis was performed by *t* test. *, *p* < 0.05; **, *p* < 0.01; ***, *p* < 0.001.

To examine the potency of ACE-oNK-HER2 to HER2^+^ cancer cells, ACE-oNK-HER2 were co-incubated with SK-OV-3 (ovarian cancer), SK-BR-3 or MCF-7 (breast cancer) cells and the cytotoxicity was measured by the xCELLigence real-time cell analysis system. As shown in Figure 2A to 2C, ACE-oNK-HER2 exhibited significantly enhanced cytotoxicity against SK-OV-3, SK-BR-3 or MCF-7 cells. It has been shown that cancer cells secrete a chemokine milieu, which leads to migration of NK cells toward tumor sites and the migration capacity is essential for NK cells to establish contact with target cells and execute anti-tumor activity [28,29]. As shown in Figure 2D, the migration of ACE-oNK-HER2 is significantly enhanced in the presence of SK-OV-3 cells.

Previous studies have reported the important role of CD107a (LAMP1) in cytolytic activity of immune cells such as NK and CD8^+^ T cells when encountering tumor cells [30,31]. The results of flow cytometry analysis showed a remarked increase of CD107a level on cell surface when ACE-oNK-HER2 was exposed to HER2^+^ cancer cells (Figure 2E and Figure S2). Furthermore, the secretion of IFNγ was significantly raised by ACE-oNK-HER2 engaging SK-OV-3 cells (Figure 2F and Figure S2). These results indicate that ACE-oNK-HER2 is activated when encountering HER2^+^ cancer cells.

**Figure 3:**
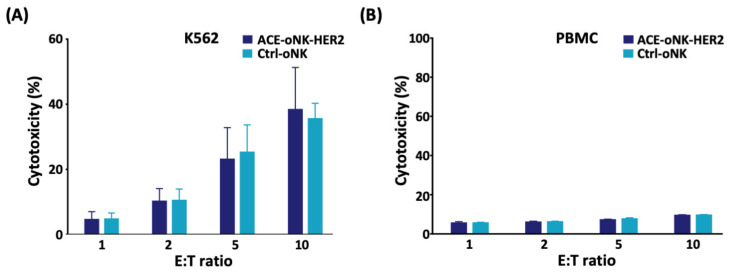
ACE-oNK-HER2 and Ctrl-oNK show no difference of cytotoxicity against HER2-negative cells. ACE-oNK-HER2 and Ctrl-oNK were co-incubated with CFSE-labeled HER2-negative (**A**) K562 human chronic myelogenous leukemia cell line or (**B**) female PBMC at effector to target a (E:T) ratio of 1:1 to 10:1. The viability of CFSE-labeled K562 and PBMC was examined using propidium iodide staining and analyzed by flow cytometry.

The specific cytotoxicity against HER2^+^ cancer cells was further evaluated by co-incubation of ACE-oNK-HER2 or Ctrl-oNK with K562 cells (HER2-negative). The results showed no significant difference of cytotoxicity between ACE-oNK-HER2 and Ctrl-oNK against K562 cells (Figure 3A), supporting the specificity of ACE-oNK-HER2 to HER2^+^ cancer cells with no change of basal cytotoxicity to HER2-negative cells. Importantly, both ACE-oNK-HER2 and Ctrl-oNK showed no compelling cytotoxicity to PBMC from healthy donors (Figure 3B). These results demonstrate that trastuzumab conjugation by ACC technology confers oNK cells with the specific cytotoxicity against HER2-expressing cancer cells.

### 2.3. Better Cytotoxicity by ACC-Mediated Trastuzumab Conjugation than ADCC against HER2^+^ Cancer Cells

**Figure 4:**
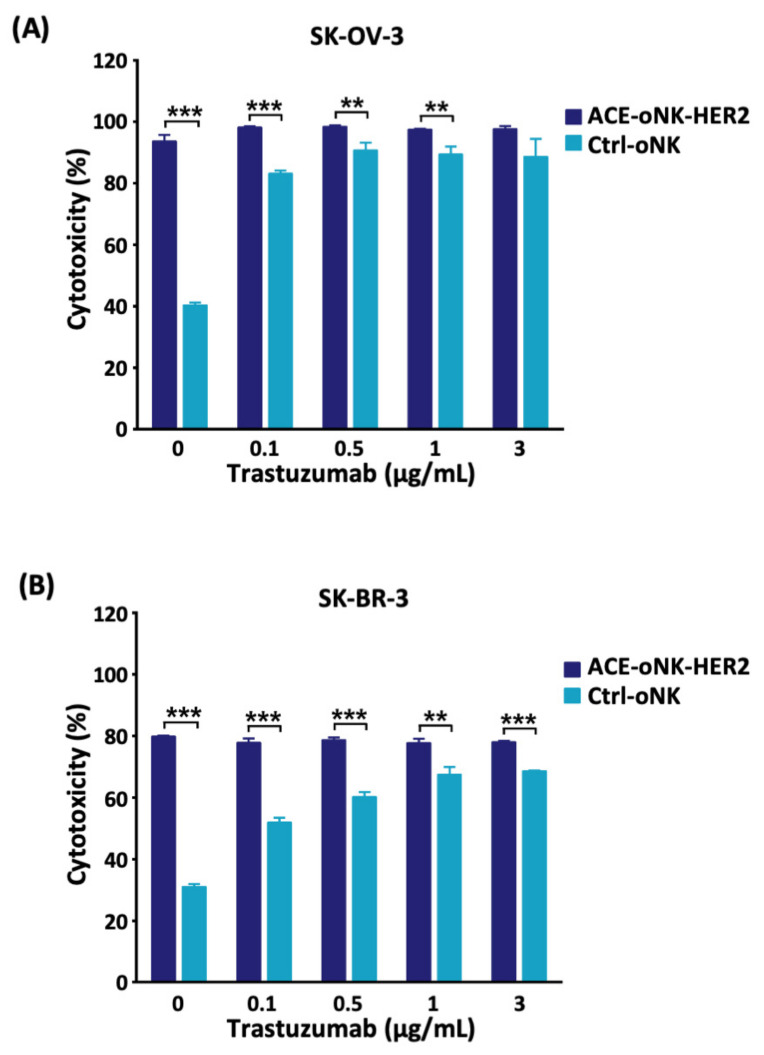
ACE-oNK-HER2 shows better cytotoxicity than ADCC of Ctrl-oNK. ACE-oNK-HER2 and Ctrl-oNK were incubated with HER2^+^ cancer cell line (**A**) SK-OV-3 and (**B**) SK-BR-3 in the presence of various trastuzumab concentrations (0, 0.1, 0.5, 1 and 3 μg/mL) in triplicate. The cytotoxicity of ACE-oNK-HER2 and Ctrl-oNK against SK-OV-3 and SK-BR-3 cells was measured and calculated by xCELLigence real-time cell analysis system. Three independent experiments were performed and the representative results were shown. Cytotoxicity was compared between treatments by *t* test. **, *p* < 0.01; ***, *p* < 0.001.

To examine the HER2-specific cytotoxicity contributed by ACC technology and ADCC, the cytotoxicity of ACE-oNK-HER2 or Ctrl-oNK were incubated with HER2^+^ cancer cells in the presence of various concentrations of trastuzumab. As shown in Figure 4, ACE-oNK-HER2 exhibited strong and consistent cytotoxicity against SK-OV-3 and SK-BR-3 cells with or without additional trastuzumab. In contrast, the specific cytotoxicity of Ctrl-oNK was enhanced by ADCC with the increased concentration of trastuzumab and reached plateau ADCC against SK-OV-3 and SK-BR-3 cells at 0.5 and 1 μg/mL of Trastuzumab, respectively (Figure 4). Of note, ACE-oNK-HER2 exhibited higher cytotoxicity than plateau cytotoxicity of Ctrl-oNK through ADCC against SK-OV-3 and SK-BR-3, suggesting that ACC-mediated trastuzumab conjugation provides oNK cells with better cytotoxicity than ADCC.

### 2.4. Reserved Potency of ACE-oNK-HER2 after Irradiation and Cryopreservation

**Figure 5:**
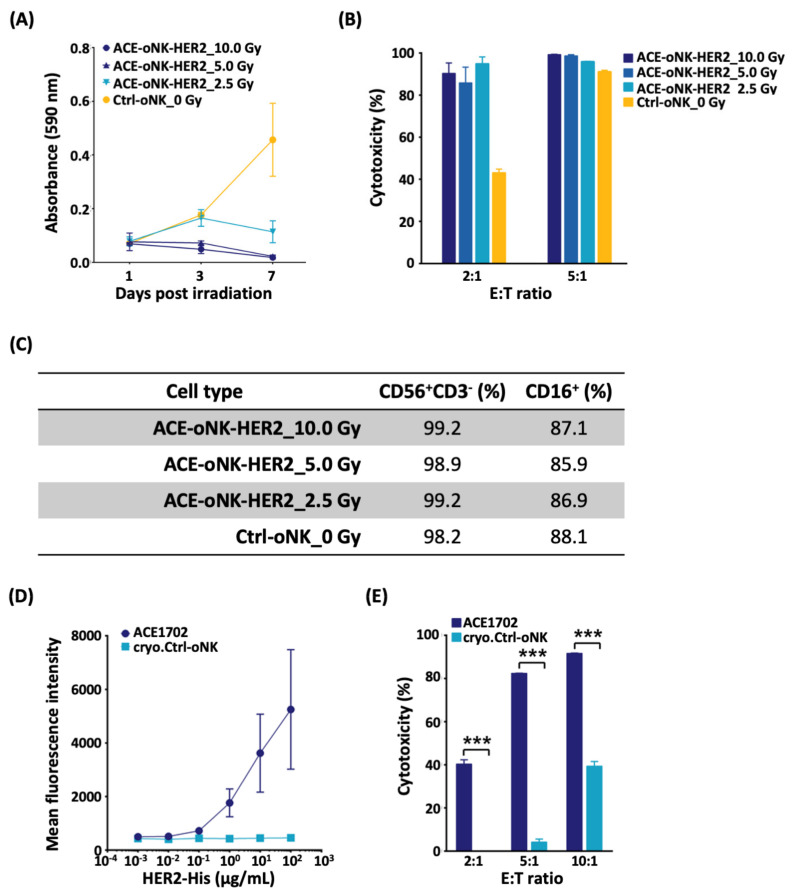
ACE-oNK-HER2 maintains the potency against HER2^+^ cancer cells after irradiation and cryopreservation. (**A**) The proliferation of ACE-oNK-HER2 irradiated at 2.5, 5.0 and 10.0 Gy as well as non-irradiated Ctrl-oNK cells were analyzed by BrdU incorporation assay. The assay was performed 1, 3 and 7 days post-irradiation. (**B**) Cytotoxicity of irradiated ACE-oNK-HER2 and non-irradiated Ctrl-oNK cells against SK-OV-3 was analyzed at an E:T ratio of 2:1 and 5:1 by the xCELLigence real-time cell analysis system. (**C**) Percentage of CD56^+^CD3^-^ and CD16^+^ population in ACE-oNK-HER2 irradiated at different doses and non-irradiated Ctrl-oNK cells was analyzed after irradiation on Day 0 by flow cytometry. (**D**) HER2 binding capacity of ACE1702 was examined by incubating with 10^−3^, 10^−2^, 10^−1^, 10^0^, 10^1^ and 10^2^ μg/mL of human HER2-His recombinant protein and HER2-bound cells were analyzed by flow cytometry. (**E**) Cytotoxicity of ACE1702 was evaluated by co-incubation with SK-OV-3 cells for 18 h. Three independent experiments were performed and the representative results were shown. Each data point was performed in triplicate. Cytotoxicity was compared between treatments by *t* test. ***, *p* < 0.001.

Previous studies suggested that γ irradiation triggers cell cycle arrest of NK cell lines without compromising their cytotoxicity and assures safety for clinical use [32,33]. To evaluate the growth arrest and potency of ACE-oNK-HER2 after irradiation, a dose titration experiment of γ irradiation of ACE-oNK-HER2 was performed. γ irradiation higher than 5 Gy can effectively triggered cell cycle arrest and eventually cell death of ACE-oNK-HER2 in 7 days (Figure 5A), while the superior cytotoxicity of ACE-oNK-HER2 was conserved with a similar surface marker profile (Figure 5B,C). Furthermore, we examined if irradiated ACE-oNK-HER2 maintained the potency to HER^+^ cancer cells after the cryopreservation. The flow cytometry-based binding assay demonstrated the HER2-binding specificity of 10 Gy-irradiated cryopreserved ACE-oNK-HER2 (ACE1702) (Figure 5D). Importantly, ACE1702 retained the migratory capacity and superior potency against SK-OV-3 cells (Figure S5 and Figure 5E). The viability and recovery rate of ACE1702 upon thawing after long-term storage had no significant variation (data not shown). These results indicate that ACE-oNK-HER2 remains potent against HER2^+^ cancer cells after 10 Gy of γ irradiation and subsequent cryopreservation.

### 2.5. In Vivo Potency of ACE1702 against HER2^+^ Ovarian Cancer Cells

**Figure 6:**
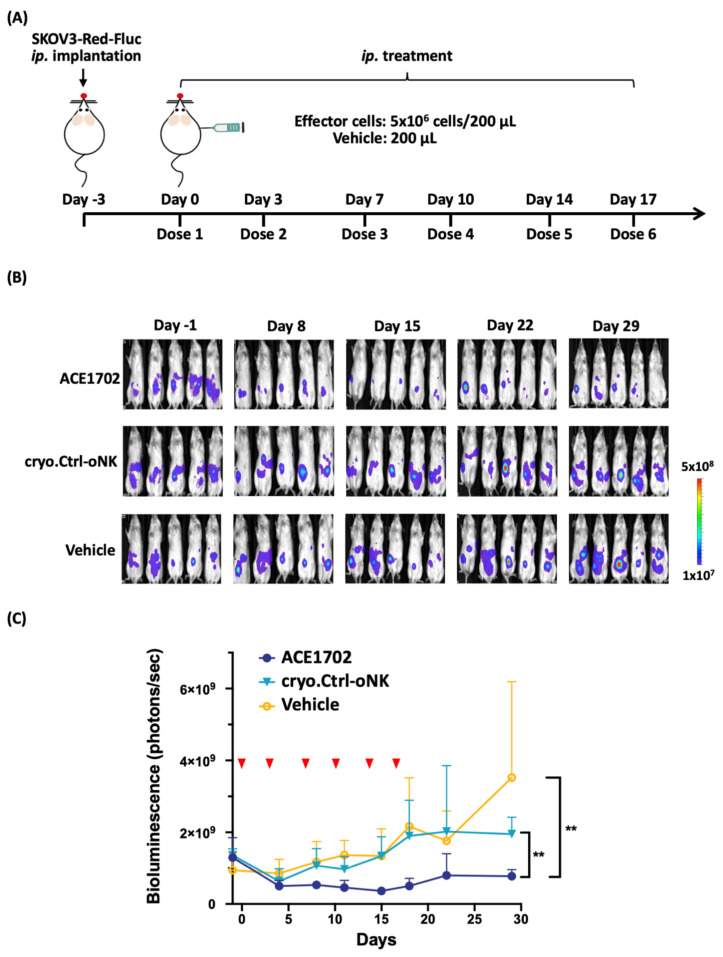
ACE1702 exhibits anti-tumor potency to HER2^+^ cancer cells in vivo. (**A**) Dosing schedule was illustrated. (**B**) The bioluminescence images of tumor burden in ACE1702-, cryo.Ctrl-oNK- and Vehicle-treated mice were captured by Ami HTX spectral instrument and analyzed by Aura software. (**C**) The bioluminescent signals of each timepoint for ACE1702-, cryo.Ctrl-oNK- and Vehicle-treated mice were presented by mean values ± SD. Red arrow heads indicate dosing of ACE1702, cryo.Ctrl-oNK and Vehicle only. Differences between 2 groups were examined by two-way ANOVA. *p* < 0.05 is considered as statistically significant. **, *p* < 0.01.

To determine the anti-cancer potency of ACE1702 in vivo, ACE1702 was intraperitoneally (*ip*.) delivered twice weekly into NSG mice *ip.* implanted with SKOV3-Red-FLuc for three weeks, as illustrated in Figure 6A. No abnormalities in clinical signs were observed throughout the study period. ACE1702-treated mice showed signs of tumor suppression after Day 8 and maintained suppression during the experimental period, whereas tumor burden was expanding in the cryopreserved γ-irradiated oNK (cryo.Ctrl-oNK)- or Vehicle-treated mice (Figure 6B). ACE1702-treated mice had a significantly lower tumor burden compared to cryo.Ctrl-oNK- and Vehicle-treated mice (Figure 6C, *p* < 0.01).

To examine the tumorigenic potential of ACE1702 and non-irradiated oNK, 1 × 10^7^ of ACE1702, non-irradiated oNK, positive control (SK-OV-3 and Daudi) and Vehicle only (DPBS) were subcutaneously implanted in female BALB/c nude mice. None (0/5) of the mice with ACE1702 and non-irradiated oNK developed any tumors throughout the study, while on Day 24 post-implantation, a tumor burden was observed in 5 out of 5 (5/5) and 4 out of 5 (4/5) mice in SK-OV-3- and Daudi-implanted groups, respectively (Table 1 and Figure S3). Additionally, we examined the persistence of ACE1702 with a single dose of *ip.* and intravenously (*iv.*) administration. The data showed that *ip.* delivered ACE1702 was localized in the peritoneal cavity, and *iv.* delivered ACE1702 remained detectable in the blood of 2 out of 5 (2/5) mice on Day 14 (Figure S4). These results demonstrate in vivo potency of ACE1702 against HER2^+^ cancer cells with no tumorigenic potential.

## 3. Discussion

We report here a novel and off-the-shelf therapeutic product ACE1702 developed from γ-irradiated CD16-expressing oNK cells. ACC platform provides oNK cells with specific cytotoxicity against HER2-expressing cancer cells. ACE1702, which is cryopreserved and γ-irradiated, still preserves the superior cytotoxicity against target cancer cells. This study demonstrates in vitro and in vivo potency of ACE1702 with no tumorigenicity, supporting its potential clinical application against HER2-expressing cancers.

CAR technology have recently been applied in NK cells though the optimal response of CAR structure combination depending on tumor types or target antigens [34]. It is noted that viral transduction and non-viral strategies are two categories to deliver CAR-expressing construct into NK cells [34,35]. The viral delivery system shows high transduction efficiency; however, a high risk of viral insertional mutation and the cost of viral vectors in clinical therapies raise concerns [36]. As for non-viral strategies, the *Sleeping Beauty* transposon system through electroporation was applied to avoid imprecise chromosomal insertion; nevertheless, the efficacy of plasmid-based, transposon-mediated gene transfer was found to be low [35]. In this study, oNK cells were conjugated with trastuzumab on cell surface with >99% conjugation efficiency using ACC technology, which is more efficient than a CD16-dependent manner (Figure 1A). Trastuzumab-armed oNK displayed specific cytotoxicity against HER2-expressing target cancer cells in vitro and in vivo (Figure 2A–C and Figure 6). Furthermore, similar basal cytotoxicity of ACE-oNK-HER2 and control oNK cells against HER2-negative K562 cell line was observed (Figure 3A), indicating HER2-binding specificity of ACE-oNK-HER2. Moreover, ACE-oNK-HER2 and Ctrl-oNK showed no significant cytotoxicity to donor PBMC cells at increasing E:T ratios (Figure 3B). No tumorigenicity was observed in ACE1702 and non-irradiated oNK (Table 1 and Figure S3). Therefore, the ACC platform provides an efficient method to arm NK cells with specific targeting capacity and superior potency without genetic engineering.

CAR-transduced NK-92 cell lines, NK-92-scFv-erbB2-CD28-ζ and NK-92/5.28.z, have been shown to exhibit cytotoxicity against HER2-expressing breast cancer cells [37,38]. In vitro cytotoxicity of NK-92/5.28.z and NK-92-scFv-erbB2-CD28-ζ cells against HER2-expressing cancer cells reached a plateau at E:T ratios of 10:1 and 20:1, respectively [37,38]. A recent study reported that conjugation of IL-2-independent NK-92MI cells with trastuzumab by α(1,3)-fucosyltransferase activity elicited cytotoxicity to breast cancer BT-474 cells at an E:T ratio of 5:1 [39]. An ACC-based trastuzumab conjugation strategy, using an efficient chemical reaction and DNA hybridization, provided oNK cells with HER2-specific binding capacity (Figure 1D). ACE-oNK-HER2 effectively eliminated HER2-expressing cancer cells at a relatively low E:T ratio (Figure 2A–C) and increased secretion of IFNγ (Figure 2F). Importantly, ACE1702, cryopreserved irradiated ACE-oNK-HER2, significantly suppressed HER2-expressing tumor burden in an ovarian cancer xenograft model, while HER2 expression of the remaining tumor mass in ACE1702-treated mice is worthy of further study (Figure 6). These results provide the evidence to support ACE1702 as a potent NK cell therapy against HER2-expressing cancers.

CAR-NK therapies have accumulated great success in clinical application, however, there are still obstacles during manufacturing of CAR-NK cells. It has been pointed out that maintaining viability and potency of the cryopreserved allogenic CAR-NK is the hurdle to fulfilling true “off-the-shelf” cell therapy [40,41]. In a previous study, Mark et al. suggested that cryopreservation may impair cytotoxicity and motility of NK cells, and the impaired cytotoxicity and motility exhibited a significant correlation [42]. The previous study indicated that cytokine-activated donor-derived NK cells were sensitive to the freeze-thaw process, and their potency were significantly reduced after thawing [43]. In contrast, NK-92 cryopreserved by a DMSO-free media maintained cytotoxic activity [44]. The recent trial of cord blood-derived NK cells further demonstrated the promising potency of NK cell therapy without serious side effects in patients [45]. In this study, trastuzumab conjugation and cryopreservation showed no significant influence on the receptor profile (Figure S1). However, the migration capability of ACE1702 was decreased (Figure S5), which might reflect the reduced potency of ACE1702 against SK-OV-3 cells in vitro and in vivo. This study demonstrates that ACE1702 reserves the NK marker profile, HER2-specific binding and both in vitro and in vivo potency (Figure 5C–E and Figure 6). Of note, intraperitoneally delivered ACE1702 was detectable in the peritoneal region for 7 days and intravenously administered ACE1702 was persistent for 14 days (Figure S4). These results suggest that ACE1702 has the potential to be an off-the-shelf NK cell therapy against HER2-expressing tumors.

Previous studies reported that trastuzumab and T-DM1 (trastuzumab-conjugated drug) revealed cytotoxic potency for breast cancer cell lines [46,47,48]. Clinical evidence further supported the efficacy and safety of T-DM1 in patients with HER2^+^ cancers [49,50]. It was noted that the higher number and ADCC activity of NK cells correlates with trastuzumab response and prolongs the survival of breast cancer patients [51]. Patients harboring CD16 with a high affinity to antibodies (i.e., CD16 158 *v/v* genotype) exhibited a good overall response rate [51]. In this study, oNK cells harboring CD16 genotype with high affinity to antibody [27] were covalently conjugated with trastuzumab through paired ssDNA linkers (Figure 1B). Our results revealed that cytotoxicity of ACE-oNK-HER2 against SK-OV-3, SK-BR-3 and MCF-7 cells was remarkably enhanced at an E:T ratio as low as 1:1 compared to Ctrl-oNK (Figure 2A–C). Trastuzumab conjugation of oNK cells by ACC platform needs notably less trastuzumab than those for ADCC and provide stronger cytotoxicity. These findings suggest that the ACC platform combined with allogeneic immune cells has the potential to provide potent and off-the-shelf therapies for cancer patients.

In summary, we applied ACC technology to develop a novel HER2-targeting NK cell product ACE1702 and provide evidence showing the in vitro and in vivo efficacy of ACE1702 against HER2-expressing cancer cells. As an economical and allogeneic NK cell therapy without genetic engineering, ACE1702 has the potential to benefit patients with HER2-expressing cancers due to its potency, affordability and off-the-shelf convenience.

## 4. Materials and Methods

### 4.1. Antibodies, Cell Lines, and Mice

Antibodies used in this study are listed in Table S1. MCF-7, SK-BR-3, SK-OV-3, Daudi and K562 were obtained from ATCC. These cell lines were cultured according to ATCC guidelines. oNK cells were cultured as described previously [27]. Female peripheral blood mononuclear cells (PBMC) were bought from PPA Research Group and cultured under the manufacturer’s instructions. Female NSG (NOD.Cg-*Prkdc^scid^ Il2rg^tm1Wjl^*/SzJ, 6–10 weeks old) and BALB/c nude mice were respectively purchased from The Jackson Laboratory (Stock No.: 005557) and BioLASCO Taiwan Co. Ltd. (Taipei City, Taiwan), and housed under the regulation of the Institutional Animal Care and Use Committee (IACUC) of the contract research organizations.

### 4.2. Antibody-Cell Conjugation Protocol

Conjugation of anti-HER2 antibody trastuzumab to oNK cells was performed as previously described [25,26]. As illustrated in Figure 1A, 5′-thiolated single-strand DNA (ssDNA), linker 1 and linker 2, were conjugated with oNK cells and trastuzumab, respectively. ssDNA-conjugated oNK and trastuzumab were co-incubated for 20 min for linker hybridization. After removing free ssDNA-conjugated trastuzumab by complete wash with Dulbecco’s phosphate-buffered saline (DPBS) (Gibco, Waltham, MA, USA), trastuzumab-linked oNK cells (ACE-oNK-HER2) were generated for further analysis.

### 4.3. Flow Cytometry Analysis

All flow cytometry analysis was performed by the Attune NxT flow cytometer installed with Attune NxT software 3.1.0. Cells were stained with fluorescent-conjugated antibodies. After being washed with DPBS twice, cell pellets were resuspended with DPBS and analyzed by flow cytometry. NK cell population was gated, and the percentage of CD56^+^CD3^−^ and anti-Fab^+^ populations were analyzed. Propidium iodide-positive (Invitrogen, USA) staining was used to determine the viability of cells.

To analyze HER2 binding capacity, cells were incubated with recombinant human HER2-His protein (ACROBiosystems, Newark, DE, USA) at designated concentration. After being washed with DPBS, the cells were then stained with FITC-conjugated anti-6X His tag antibody. Stained cells were washed by DPBS and analyzed by flow cytometry.

ACE-oNK-HER2 was co-incubated with SK-OV-3 cells at E:T ratio of 1:1 at 37 °C, 5% CO_2_ in the presence of brefeldin A (4 μg/mL) (BioLegend, San Diego, CA, USA) and monensin (1 μM) (BioLegend, USA) for 4 h. ACE-oNK-HER2 alone was incubated at the same condition as cytokine spontaneous release control. Effector cells were harvested and washed. The staining of degranulation marker CD107a and intracellular cytokine IFNγ was performed according to the manufacturer’s protocol of Fix and Perm^®^ Cell Permeabilization Kit (ThermoFisher, Waltham, MA, USA). The fold change of CD107a and IFNγ expression in ACE-oNK-HER2 co-cultured with SK-OV-3 cells was calculated relative to ACE-oNK-HER2 alone (set as 1).

### 4.4. In Vitro Cytotoxicity

The xCELLigence real-time cell analysis system was applied under the manufacturer’s instruction. In brief, HER2^+^ cancer cell line MCF-7, SK-BR-3 and SK-OV-3 were seeded into wells of E-plate and incubated to settle cell attachment. Trastuzumab-conjugated or control oNK cells and various concentrations of trastuzumab (0, 0.1, 0.5, 1 and 3 μg/mL) were added into wells with HER2^+^ cancer cells. The cell index (CI) was recorded throughout the study. In the absence of target cells, CI of wells containing effector cells was regarded as an effector background. The cytotoxicity was calculated by xCELLigence RTCA software automatically.

K562 and PBMC from female donor were stained with carboxyfluorescein succinimidyl ester (CFSE) at a final concentration of 5 μM for 10 min at room temperature. CFSE-stained K562 or PBMC cells were cultured with Ctrl-oNK or ACE-oNK-HER2 at an E:T ratio of 1:1 to 10:1 for 4 h. The cell mixture was washed by DPBS once and stained with propidium iodide. The viability of CFSE-stained K562 and PBMC (CFSE^+^PI^-^) was analyzed by the Attune NxT flow cytometer.

### 4.5. Migration Assay

To examine migration capacity, cells were seeded into the cell culture insert (Millipore, Burlington, MA, USA) with a 3 μm pore size. The effector-containing culture inserts were immersed in wells in the absence and presence of SK-OV-3 cells. After 21 h incubation at 37 °C in 5% of CO_2_, migratory cells were harvested and stained with FITC-conjugated anti-human CD56 antibody for flow cytometry analysis. Total CD56^+^ cells were recorded as migrated cells.

### 4.6. BrdU Assay

Effector oNK cells irradiated at 2.5, 5.0 and 10.0 Gy were conjugated with trastuzumab. Non-irradiated oNK was regarded as Ctrl-oNK cells. The assay was performed according to the manufacturer’s manual (Abcam, Cambridge, UK). In brief, irradiated ACE-oNK-HER2 and non-irradiated Ctrl-oNK cells were seeded in each well of a 96-well plate. BrdU stock was diluted to working concentration and added to the corresponding wells one day before sampling on Day 1, 3 and 7. On the sampling day, cells were fixed, and then the BrdU incorporated into DNA was detected by the anti-BrdU antibody. The wells containing cells without BrdU incubation serve as the background. The ELISA signals generated through an HRP-conjugated secondary antibody-mediated colorimetric method.

### 4.7. Animal Studies

For evaluation of in vivo efficacy, 3 × 10^5^ of SKOV3-Red-FLuc (Perkin Elmer, Waltham, MA, USA) cells were *ip.* implanted into female NSG mice three days before treatment. Five tumor-bearing mice per group were treated with 5 × 10^6^ of ACE1702, corresponding Ctrl-oNK and vehicle on Day 0, 3, 7, 10, 14 and 17. Tumor luminescence signals were captured on Day -1, 4, 8, 11, 15, 18, 22 and 29 by Ami HTX spectral instrument (Accela, Prague, Czech) and analyzed by Aura software. All mice were sacrificed on Day 29 after luminescence image capture, and necropsy was performed.

For evaluation of in vivo tumorigenic potential, 1 × 10^7^ of ACE1702, non-irradiated oNK, SK-OV-3 and Daudi in 100 μL of DPBS were subcutaneously implanted into female BALB/c nude mice according to previous study design with some modification [32]. An equal volume of DPBS was implanted as a negative control. Five mice were included in each group. Tumor size was measured by caliber, and the corresponding volume was calculated by length × width × width × 0.52. The study duration was 60 days.

### 4.8. Statistical Analysis

The data of cytotoxicity assay and HER2 recombinant protein binding capacity assay were analyzed with paired or unpaired Student’s *t* tests. Animal studies were analyzed with two-way ANOVA. *, *p* < 0.05; **, *p* < 0.01 and ***, *p* < 0.001.

## 5. Conclusions

In summary, we applied ACC technology with oNK cell line to develop a novel HER2-targeting NK cell therapy ACE1702. With no tumorigenic potential, ACE1702 was shown to be superior in vitro and in vivo potency against HER2-expressing cancer cells. As an economical and allogeneic NK cell therapy without genetic engineering, ACE1702 has the potential to benefit patients with HER2-expressing cancers through its potency, affordability and off-the-shelf convenience.

## Figures and Tables

**Table 1:** Summary of tumor incidence.

Cell Type	Tumor Incidence				
	Day 14	Day 21	Day 24	Day 42	Day 59
ACE1702	0/5	0/5	0/5	0/5	0/5
Non-irradiated oNK	0/5	0/5	0/5	0/5	0/5
SK-OV-3	5/5	5/5	5/5	5/5	5/5
Daudi	0/5	3/5	4/5	4/5	4/5
DPBS	0/5	0/5	0/5	0/5	0/5

## Data Availability

The data presented in this study are openly available in Supplementary Materials.

## Supplementary Materials

The following are available online at https://www.mdpi.com/article/10.3390/cancers13112724/s1. Figure S1: The expression of CD56, CD16, NKp30, NKp44, NKG2A and killer-cell immunoglobulin receptors (KIRs) in ACE1702 and cryo.Ctrl-oNK. FITC-anti-TCRVd2 antibody was used as negative target staining of CD56. PE/Cy7-anti-CD4 antibody was used as negative target staining of CD16, NKp30 and NKp44. PE-anti-CD8 antibody was used for negative target staining of NKG2a and pan-KIR. Shaded peaks showed corresponding staining of negative targets. Figure S2: Flow cytometry analysis of CD107a and IFNγ levels in ACE-oNK-HER2 incubated with or without SK-OV-3 cells. APC-Cy7-conjugated antibody against human TCRVd2 and PE/Cy7-conjugated against human CD4 were used for negative target staining of CD107a and IFNγ, respectively. Figure S3: No tumorigenicity of ACE1702 and oNK cells in vivo. 1 × 107 of ACE1702, non-irradiated oNK, SK-OV-3 and Daudi were subcutaneously implanted into female BALB/c nude mice. Five mice were included in each group and tumor size were monitored for 60 days. Representative images of mice implanted with ACE1702, non-irradiated oNK, SK-OV-3 and Daudi were shown. Tumors were indicated by black arrowheads. Figure S4: Detection of ACE1702 in the peritoneum and blood. (A) 5 × 106 of ACE1702 was intraperitoneally delivered into BALB/c nude mice (4 mice per timepoint). Viable CD56+Fab+ cells in peritoneal lavage were stained with anti-human CD56 antibody and anti-F(ab’)2 antibody and detected by flow cytometry on Day 0, 1, 3, 5, 7 and 14. (B) 3 × 106 of ACE1702 was intravenously delivered into BALB/c nude mice (5 mice per timepoint). ACE1702 was stained with anti-human CD56 antibody and anti-F(ab’)2 antibody and detected by flow cytometry on Day 0, 1, 3, 5, 7 and 14; Figure S5: Migratory capacity of ACE1702. The migrated ACE1702 in the absence and presence of SK-OV-3 were harvested and stained with anti-CD56 antibody for flow cytometry analysis. Table S1: List of antibodies used in this study; Supplementary Methods.
