# Supplementary material for: A Novel off-the-Shelf Trastuzumab-Armed NK Cell Therapy (ACE1702) Using Antibody-Cell-Conjugation Technology
Source: Cancers (Basel). 2021 May 31;13(11):2724. doi: 10.3390/cancers13112724 (PMC8199224; doi:10.3390/cancers13112724)
Supplement: Supplementary file 1 [file cancers-13-02724-s001.zip › cancers-1213792-supplementary.pdf]

A Novel Off-the-Shelf Trastuzumab-Armed NK Cell Therapy (ACE1702) Using Antibody-Cell-Conjugation Technology

Hao-Kang Li *, Ching-Wen Hsiao, Sen-Han Yang, Hsiu-Ping Yang, Tai-Sheng Wu, Chia-Yun Lee, Yan-Liang Lin, Janet Pan, Zih-Fei Cheng, Yan-Da Lai, Shih-Chia Hsiao and Sai-Wen Tang *

Supplementary Table:

Table S1. List of antibodies used in this study. *, the anti-human pan-KIR antibody detects human cells transfected with KIR2DL2, 2DL3, 2DS2, or 2DS4, but not cells transfected with KIR2DL1, 2DL4, 3DL1, or 3DL2 as described in manufacturer's specification.

Name	Catalog number	Vendor
**Phycoerythrin-AffiniPure Goat Anti-Human IgG, F(ab')2 Fragment Specific**	109-116-097	Jackson Immuno Research Labs
**FITC- anti human CD56 antibody**	318304	BioLegned
**PE/Cy5 anti-human CD3 antibody**	300410	BioLegend
**PE/Cy7 anti-human CD16 antibody**	302016	BioLegend
**APC/Cy7 anti-human CD107a antibody**	328630	BioLegend
**FITC anti-human TNFα antibody**	502906	BioLegend
**PE/Cy7 anti-human IFNγ antibody**	502528	BioLegend
**PE/Cy7 anti-human NKp30 antibody**	325214	BioLegend
**PE/Cy7 anti-human NKp44 antibody**	325116	BioLegend
**PE anti-human NKG2A antibody**	FAB1059P	R&D Systems
**PE anti-human pan-KIR antibody***	FAB1848P	R&D Systems
**FITC anti-human TCRVd2 antibody**	331406	BioLegend
**PE anti-human CD8 antibody**	344706	BioLegend
**PE/Cy7 anti-human CD4 antibody**	357410	BioLegend
**APC/Cy7 anti-human TCRVd2 antibody**	331440	BioLegend

Supplementary Figures

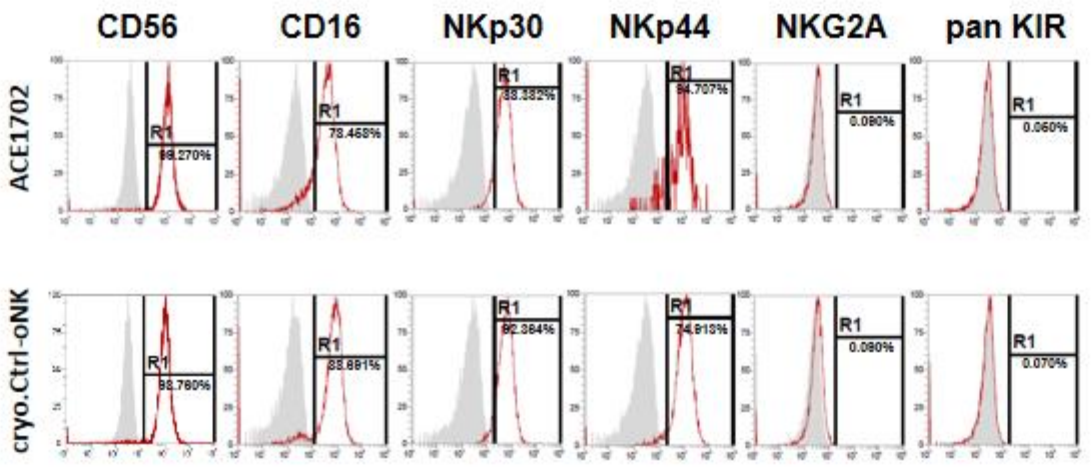

Figure S1. The expression of CD56, CD16, NKp30, NKp44, NKG2A and killer-cell immunoglobulin receptors (KIRs) in ACE1702 and cryo.Ctrl-oNK. FITC-anti-TCRVd2 antibody was used as negative target staining of CD56. PE/Cy7-anti-CD4 antibody was used as negative target staining of CD16, NKp30 and NKp44. PE-anti-CD8 antibody was used for negative target staining of NKG2a and pan-KIR. Shaded peaks showed corresponding staining of negative targets.

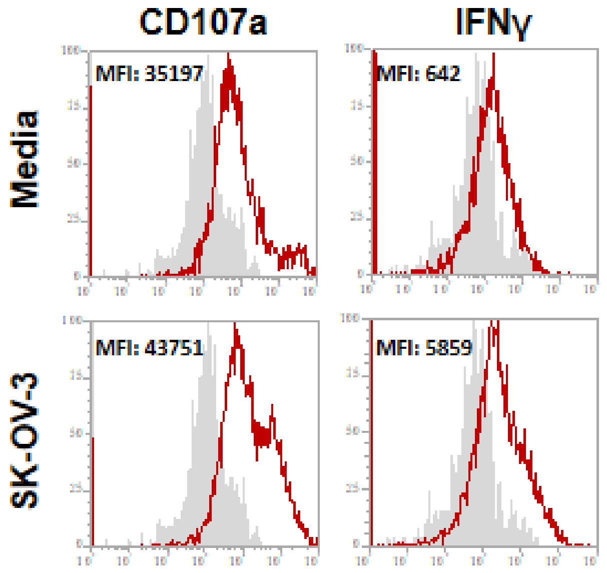

Figure S2. Flow cytometry analysis of CD107a and IFNγ levels in ACE-oNK-HER2 incubated with or without SK-OV-3 cells. APC-Cy7-conjugated antibody against human TCRVd2 and PE/Cy7-conjugated against human CD4 were used for negative target staining of CD107a and IFNγ, respectively.

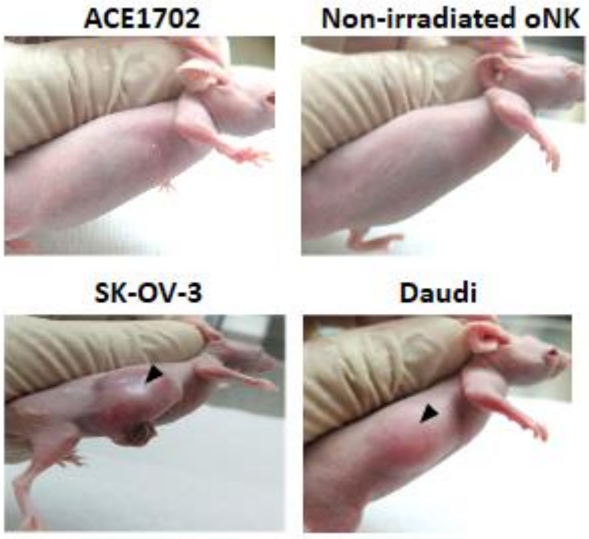

Figure S3. No tumorigenicity of ACE1702 and oNK cells in vivo. 1×10^7 of ACE1702, non-irradiated oNK, SK-OV-3 and Daudi were subcutaneously implanted into female BALB/c nude mice. Five mice were included in each group and tumor size were monitored for 60 days. Representative images of mice implanted with ACE1702, non-irradiated oNK, SK-OV-3 and Daudi were shown. Tumors were indicated by black arrowheads.

(A)

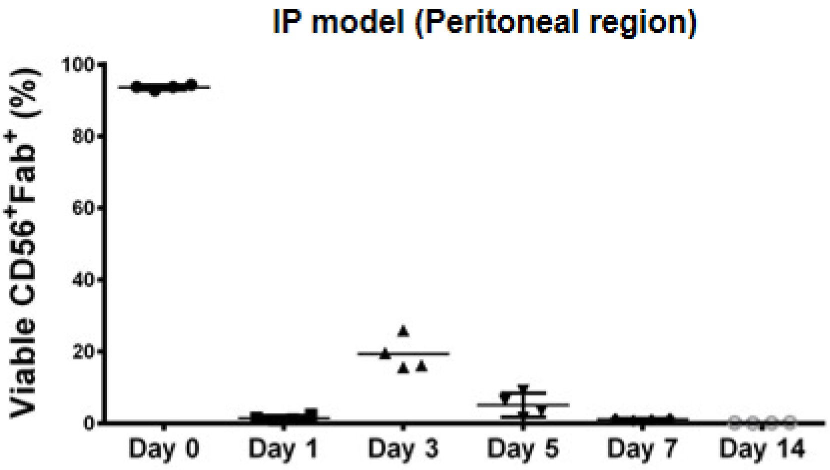

(B)

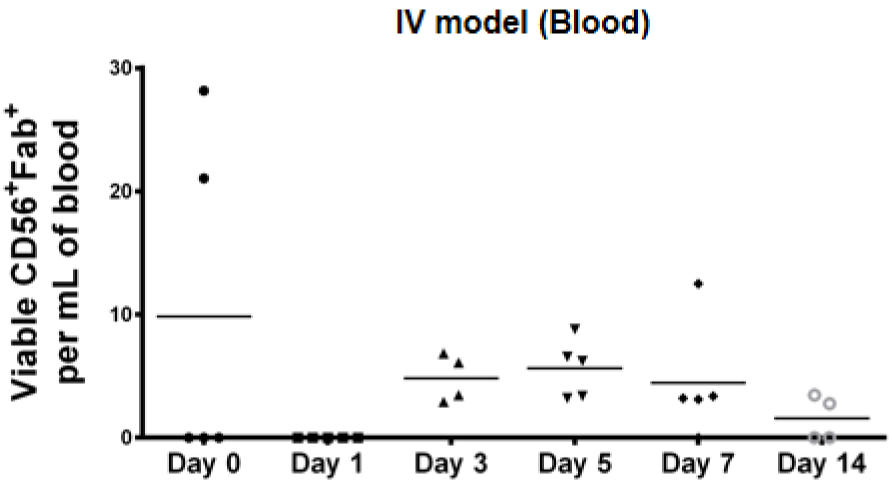

Figure S4. Detection of ACE1702 in the peritoneum and blood. (**A**) 5×10^6 of ACE1702 was intraperitoneally delivered into BALB/c nude mice (4 mice per timepoint). Viable CD56+Fab+ cells in peritoneal lavage were stained with anti-human CD56 antibody and anti-F(ab')2 antibody and detected by flow cytometry on Day 0, 1, 3, 5, 7 and 14. (**B**) 3×10^6 of ACE1702 was intravenously delivered into BALB/c nude mice (5 mice per timepoint). ACE1702 was stained with anti-human CD56 antibody and anti-F(ab')2 antibody and detected by flow cytometry on Day 0, 1, 3, 5, 7 and 14;.

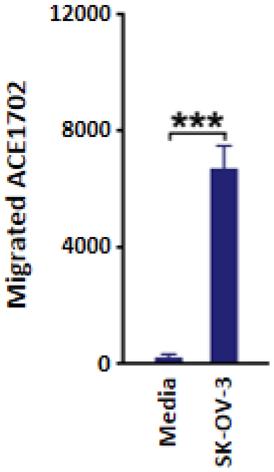

Figure S5: Migratory capacity of ACE1702. The migrated ACE1702 in the absence and presence of SK-OV-3 were harvested and stained with anti-CD56 antibody for flow cytometry analysis.
